# Multicellular String-Like Structure Formation by *Salmonella* Typhimurium Depends on Cellulose Production: Roles of Diguanylate Cyclases, YedQ and YfiN

**DOI:** 10.3389/fmicb.2020.613704

**Published:** 2020-12-14

**Authors:** Alan Varghese, Semanti Ray, Taru Verma, Dipankar Nandi

**Affiliations:** ^1^Undergraduate program, Indian Institute of Science, Bengaluru, India; ^2^Department of Biochemistry, Indian Institute of Science, Bengaluru, India; ^3^Centre for Biosystems science and engineering, Indian Institute of Science, Bengaluru, India

**Keywords:** *Salmonella*, multi-cellularity, cellulose, c-di-GMP, stress responses

## Abstract

Bacteria face diverse stresses in the environment and, sometimes, respond by forming multi-cellular structures, e.g., biofilms. Here, we report a novel macroscopic and multi-cellular structure formed by *Salmonella* Typhimurium, which resembles small strings. These string-like structures, ∼1 cm long, are induced under some stress conditions: iron deprivation by 2,2-Bipyridyl or low amounts of antibiotics or ethanol in minimal media. However, cells in strings revert back to planktonic growth upon return to nutrient rich media. Compared to planktonic cells, strings are more resistant to antibiotics and oxidative stress. Also, strains lacking *csgD* or *rpoS*, which are defective in the classical rdar biofilm formation, form strings. Furthermore, some biofilm inducing conditions do not result in strings and vice-versa, demonstrating that strings are not related to classical CsgD-dependent biofilms. Cells in a string are held together by cellulose and a strain lacking *bcsA*, which is defective in cellulose production, does not form strings. In addition, reductive stress conditions such as dithiothreitol (DTT) or mutations in the Disulfide bonding system (DSB) also give rise to strings. The amounts of c-di-GMP are increased upon string formation and studies with single and double deletion strains of the diguanylate cyclases, *yedQ* (STM1987) primarily and *yfiN* (STM2672) partly, revealed their importance for string formation. This is the first study showcasing the ability of *Salmonella* to produce high amounts of cellulose in liquid culture, instead of an interface, in a CsgD-independent manner. The relevance and possible applications of strings in the production of bacterial cellulose and bioremediation are discussed.

## Introduction

Bacteria constantly assess their environment for nutrient availability, presence of chemical messengers, stress-inducing chemicals etc. These cues dictate bacterial responses, resulting in changes in morphology at a single cell level or forming multi-cellular structures. (Galperin, 2018). An array of bacterial multi-cellular structures has been described, including those made by filamentous bacteria e.g., *Candidatus*, *Mycobacterial* cording in detergent free media, aggregates of *Zymomonas* in minimal media, clumping behavior by certain *Rhodobacter* mutants, to the most well studied ones like biofilms which could be composed of a single or multiple species (Puskas et al., 1997; Shapiro, 1998; Rudolph et al., 2001; Bernut et al., 2014; Lyons and Kolter, 2015; Jones-Burrage et al., 2019). Multi-cellular structures, the most well studied being biofilms, can be induced by a myriad of stresses including differences in temperature, osmolarity, nutrient availability etc. (Stanley and Lazazzera, 2004; Ray et al., 2020). Sub-Minimum Inhibitory Concentration (MIC) levels of antibiotics are also known to induce biofilms. Biofilms are known to be more resistant to antibiotics (up to a 1000-fold) and other stresses over planktonic cells by forming a barrier, reducing cellular activity, increasing expression of multiple drug resistance pumps, expression of glycans that trap antibiotics etc. (Mah and O’Toole, 2001; Mah et al., 2003; Høiby et al., 2010; Kaplan, 2011).

*Salmonella* Typhimurium, an intracellular pathogen, is known to form robust biofilms at interfaces/surfaces in lab settings as well as inside the host gall bladder, where it resides during the carrier state (Mireles et al., 2001; Crawford et al., 2010; Pontes et al., 2015). Cells in the *Salmonella* biofilms are held together by various proteinaceous components like curli fimbriae, exopolysaccharides like cellulose, and others like colanic acid, enterobacterial common antigen, O-antigen capsule, extracellular DNA (Pontes et al., 2015). Also, *S.* Typhimurium biofilm formation is controlled by the bacterial secondary messenger c-di-GMP and the transcription factor CsgD, which control production of various extracellular polymers as well as quorum sensing molecules (Xavier and Bassler, 2003; Parsek and Greenberg, 2005; Ahmad et al., 2017). However, a CsgD-independent Type 1 fimbriae mediated biofilm formation has also been reported (Monteiro et al., 2012). The two major extracellular components of *Salmonella* biofilms are curli fimbriae and cellulose. The production of cellulose depends on the cellulose synthase complex, consisting of members of the *bcs* operon and c-di-GMP which allows the cellulose synthase complex to become active (Römling and Galperin, 2015). Levels of c-di-GMP are in turn controlled by GGDEF-domain containing diguanylate cyclases (DGCs) which produce c-di-GMP and EAL or HD-GYP domain containing phosphodiesterases (PDEs) which degrade c-di-GMP (Simm et al., 2014). *Salmonella* has been reported to produce cellulose in multiple conditions such as low osmolarity in rich medium, in nutrient deficient medium such as ATM and inside macrophages (Römling, 2002; Stanley and Lazazzera, 2004; Pontes et al., 2015; Römling and Galperin, 2015). Recent studies in *E. coli* and *Salmonella* have, however, shown that cellulose production may occur in a manner independent of transcription factor CsgD via DGC YedQ or under reductive stresses via YfiN (Da Re and Ghigo, 2006; Grantcharova et al., 2010; Hufnagel et al., 2014; Römling and Galperin, 2015; Ahmad et al., 2016). Notably, cellulose production represses bacterial virulence (Pontes et al., 2015; Römling and Galperin, 2015). Cellulose produced by *E. coli* and *Salmonella* Typhimurium is a modified form, wherein alternate glycosyl residues in the cellulose carry a phosphoethanoloamine group (Galperin and Shalaeva, 2018; Thongsomboon et al., 2018). Interestingly, bacterial cellulose is a material of great biomedical significance and cellulose produced by *Gluconacetobacter hanseni* is presently being used for burn wound dressings. Bacterial cellulose is suitable for this due to properties such as high-water retention capacity, low toxicity, low immunogenicity and ease of purification compared to plant cellulose (Fu et al., 2013; Portela et al., 2019).

Apart from swarming motility and classical biofilms, group behavior/structures in *Salmonella* have not been reported extensively. Our lab has studied the various genetic and environmental factors that affect biofilm formation and stress responses by *Salmonella* (Kumar and Nandi, 2007; Bhosale et al., 2013; Ray et al., 2019; Ray et al., 2020; Thakur et al., 2020). While studying some stress responses, we observed macroscopic string-like structures. In this study, we report this novel string-like multi-cellular structure dependent on cellulose in *Salmonella* Typhimurium that is induced under certain stress conditions, e.g., iron deprivation by 2,2 –Bipyridyl (Bipd), Dithiothreitol (DTT), sub-MIC amounts of antibiotics in minimal media. We find that this macroscopic structure is not related to classical CsgD-dependent biofilms and confers increased resistance to various stresses compared to planktonic cells. We also report that high amounts of c-di-GMP are found in strings and the diguanylate cyclases, *yedQ* primarily and *yfiN* partly, are important for formation of strings.

## Materials and Methods

### Bacterial Strains and Growth Conditions

The bacterial strains used in this study are listed in Table 1. All cultures were grown in Luria-Bertani (LB) medium consisting of 10 g of tryptone (Himedia, Mumbai, India), 10 g of NaCl (Merck, Mumbai, India) and 5 g of yeast extract (Himedia) per liter at 37°C with constant shaking at 180 rpm. Single colony cultures grown for 8 h-10 h served as pre-inoculum cultures for all experiments. Antibiotics were used at 30 μg/ml chloramphenicol, 50 μg/ml of streptomycin (Sigma, United States), 100 μg/ml ampicillin (Himedia). Single gene knockouts were generated using one step chromosomal gene inactivation as described using plasmids and primers in Tables 1 and 2 (Datsenko and Wanner, 2000). The antibiotic resistance cassette was then removed using pCP20 before being used.

**TABLE 1:** Strains used and relevant characteristics.

Strain	Relevant characteristics (with resistance cassette if any)	Source
Escherichia coli DH5α	Strain used for generation and propagation of plasmids, endA, hsdR17, supE44, thi-1, recA1 gyrA, relA1, Δ(lacZYA-argF)U169, deoR (Φ80dlacΔ(lacZ)M15)	Kumar and Nandi, 2007
**Salmonella enterica serovar Typhimurium**		
14028s	Wild type strain for study	Kumar and Nandi, 2007
ΔbcsA	ΔbcsA in 14028s background	This study
ΔadrA	CmR, ΔadrA in 14028s background	This study
ΔcsgD	CmR, ΔcsgD in 14028s background	This study
ΔrpoS	CmR, ΔrpoS in 14028s background	This study
ΔdsbA	ΔdsbA in 14028s background	This study
ΔyedQ	ΔyedQ in 14028s background	This study
ΔyfiN	KanR, ΔyfiN in 14028s background	This study
ΔyedQΔyfiN	KanR, ΔyedQΔyfiN in 14028s background	This study
SL 1344	His auxotroph	Subramanian and Qadri, 2006
Pseudomonas aeruginosa PA10	Wild type	Holloway and Morgan, 1986
Escherichia coli MG1655	Wild type	Kumar and Nandi, 2007
**Plasmids**		
pKD46	AmpR, Arabinose inducible λ red recombinase	Datsenko and Wanner, 2000
pKD3	AmpR, FRT CmR FRT	Datsenko and Wanner, 2000
pCP20	AmpR, CmR, FLP Recombinase,	Datsenko and Wanner, 2000
pTrc99a	AmpR, Empty vector for cloning	Ray et al., 2020
pbcsA	AmpR,WT bcsA gene from 14028s cloned between SacI and XbaI in ptrc99a	This study
pPROEX Htb GFP	AmpR, IPTG inducible GFP expression	Ray et al., 2020
pPROEX Htb mCherry	AmpR, IPTG inducible mCherry expression	Ray et al., 2020

**TABLE 2:** Primers used in the study and their purpose.

Primer	Sequence (5′ – 3′)	Purpose
bcsA-KO-FP	GCGGGCGACAAAACGTCCGCCGGGAGCCTG CGATGGTGTAGGCTGGAGCTGCTTCG	bcsA deletion
bcsA-KO-RP	TCCAGGACAATTTTCTTTTCATCGCATT ATCATCACGGCTGACATGGGAATTAGCCATGGTCC	bcsA deletion
csgD-KO_FP	GGGCAGCTGTCAGATGTGCGATTAAAAAA AGTGGAGTTTCATCGTGTAGGCTGGAGCTGCTTCG	csgD deletion
csgD-KO_RP	CAATCCAGGTCAGATAGCGTTTCATGGCCTTAC CGCCTGCGGCTGACATGGGAATTAGCCATGGTCC	csgD deletion
rpoS-KO_FP	CAGGCTTTGACTTGCTAGTTCCGTCAAGGGATCAC GGGTAGGTGTAGGCTGGAGCTGCTTCG	rpoS deletion
rpoS-KO_RP	GGCCAGTCGACAGACTGGCCTTTTTTTGACAAGGG TACTTACGGCTGACATGGGAATTAGCC	rpoS deletion
bcsA-Cloning-FP	AGACGAGCTCATCCGGGAGCCTGCGATGAGCG CCCTTTCCCG	Generation of bcsA:AmpR
bcsA-Cloning-RP	CTAGTCTAGATCATTTATCGTCATCGTCTTTGT AATCTTGTTGAGCCTGAGCCATAACCCGATC	Generation of bcsA:AmpR
dsbA-KO-FP	TACAATTAACGCCAATGTATTAATCGGAGAGAGTT GATCATGGTGTAGGCTGGAGCTGCTTCG	dsbA deletion
dsbA-KO-RP	ACATCTTATAAAAACGCCGGTCAGTGACCGGCGTT CTTTTTACGGCTGACATGGGAATTAGCC	dsbA deletion
adrA-KO-FP	CCATGCGCTCTGTTTCTATAATTTGGGAAAATTGT TTCTAAATGGTGTAGGCTGGAGCTGCTTCG	adrA deletion
adrA-KO-RP	TCAGAGGCGCTCAGTAAATCCTGAAGCCCGGCTG GACGTCACGGCTGACATGGGAATTAGCC	adrA deletion
yedQ-KO-FP	TGGCTACCGTAAGCCATCAGGGGGAGTTGTATCAA TAACCAGGAGTGTAGGCTGGAGCTGCTTCG	yedQ deletion
yedQ-KO-RP	AGCCAGAACGAAGGGCCGGATGGCTGGCGCGAAG GAATGGACTACGGCTGACATGGGAATTAGCC	yedQ deletion
yfiN-KO-FP	AATCCAGAAGTATTAATGCTTGCACGGAATCAAAA GCATGGCCATGGTCCATATGAATATCCTCC	yfiN deletion
yfiN-KO-RP	GTCTCAACGCTGAGTCAGAAACGGCCAGGCCCGTT CCTTAGTGTAGGCTGGAGCTGCTTC	yfiN deletion
dsbA-RT-FP	TCCTTCTACTGCCCACATTG	qRT-PCR
dsbA-RT-RP	CTTCTACACCCAACGCCATC	qRT-PCR
rrlC-RT-FP	GAGCGTTCTGTAAGCCTGTG	qRT-PCR
rrlC-RT-RP	CGCAGTAACACCAAGTACGG	qRT-PCR

### Single and Double Gene Knockout Construction

Single and double gene knockouts were generated using one step chromosomal gene inactivation as previously described using plasmids and primers in Tables 1 and 2 (Datsenko and Wanner, 2000). Double knockouts were constructed after the removal of the antibiotic resistance cassette from the single gene knockouts (Ray et al., 2019). The antibiotic resistance cassette was then removed using pCP20 before being used.

### String Formation

M9 minimal medium was made as per the Cold Spring Harbor Laboratory protocol: 5X salt solution (per liter: 33.92 g Na_2_HPO_4_, 15 g KH_2_PO_4_, 5 g NH_4_Cl, all from Fisher Scientific, Mumbai, India) 2.5 g NaCl (Merck), 2 mM MgSO_4_ (Fisher Scientific), 100 μM CaCl_2_ (Merck), 4 g Glucose (Himedia). To induce strings unless mentioned 200 μM of 2,2-Bipyridyl (Bipd) (Sigma) was added to M9 minimal medium. Strings were harvested using sterile cut-tips after 10 hours at 37 °C with constant shaking at 180 rpm and processed appropriately. Planktonic growth was measured as optical density at 600 nm.

### String Quantitation

Strings were washed thoroughly twice with sterile PBS to remove planktonic cells and were stained using 50 μl of 2.5 μM of SYTO 9 (Molecular Probes, OR, United States), followed by incubation at room temperature at 90 rpm for 3 hours. The fluorescence intensity was measured with 488 nm excitation and 528 nm emission wavelength (Römling and Galperin, 2015) using Tecan Infinite PRO 200 (Austria). Due to fluctuations in laser intensity during some experiments involving M9 + Bipd, fluorescence values in those experiments were normalized such that the average WT fluorescence in M9 + Bipd was set at 10000 a.u.

### Cellulose Staining

Both strings and planktonic cells were harvested at 10 h and washed with PBS. They were then placed on a glass slide and 100 μl of 100 μg/ml Fluorescent Brightener 28 solution (pH 10) (Sigma) was added and incubated for 5 min followed by washing to remove excess stain before confocal imaging. For Calcofluor staining on LB minus NaCl agar plates, 20 μg/ml of Calcofluor white was added (Fluka, Canada).

### CFSE staining

Strings approximately 0.5 cm were isolated at 6-hour time point from M9 + 200 μM 2,2- Bipyridyl and washed with PBS once. Strings were stained with 2.5 μM of CFSE (Sigma) in PBS and kept for 90 minutes at 37°C and 180 rpm. The excess stain was washed using PBS. The washed strings were inoculated in fresh M9 + 200 μM 2,2- Bipd + Glu for specified time points and in M9- Glu + 200 μM 2,2- Bipd for the remaining time that would add up to 18 hours at 37°C and 180 rpm. Strings were harvested after 18 hours and prepared for confocal imaging.

### Antibiotic and H_2_O_2_ Treatment of Strings and Cells

Planktonic cells and strings at 10-hour time points were taken for the experiment. 50 μl of 2 OD culture or strings roughly 1 cm in length were added to M9 medium for 3 h at 37°C and 180 rpm. The viability of planktonic cells was determined using CFU enumeration on SS Agar plates after plating appropriate dilutions. CFU/ml was then compared among various conditions to estimate survival. CFU was used for planktonic cells, since single cells would most likely get lysed due to ampicillin and kanamycin treatments.

The viability of cells in a string was determined using Propidium Iodide staining. Strings were washed twice with PBS and stained with 15 μM of PI for 20 min at 37°C and 180 rpm. The excess PI was removed by gentle washing.

To calculate the viability of strings (V_T_) following treatment ‘T’, the following equation was used,

VT={FD−FT(FD−FM9)}∗100

where, *F*_*T*_ is Mean Fluorescence Intensity (MFI) of PI stained strings post treatment T, *F*_*D*_ is MFI of dead strings and *F*_*M*9_ for strings kept in M9 alone.

To obtain the formula, we assumed that the all cells in the strings kept in M9 alone were viable, leading to F_M__9_ being the background signal due to basal auto-fluorescence or small amount of residual stain after washing. We have also taken F_D_ to be the maximum MFI, since all cells in a string treated with Isopropanol were dead (by observing no growth after adding washed isopropanol treated strings into fresh LB, data not shown). Thus, F_D_-F_M__9_ represents the difference in MFI when all cells are either dead or alive. Any intermediate survival reflected by F_T_ can be translated to percentage viability by removing the fluorescence due dead cells shown by F_T_.

### Formation of Multi-Species Strings

*E.coli* (MG1655) was transformed with (pProEX HTb mCherry) and *S*. Typhimurium with (pProEX HTb GFP). Pre-inoculums were made with LB medium containing 100 μg/ml ampicillin and 400 μM IPTG. The bacteria were mixed in 1:1 ratio amounting to a total of 10^8 cells in total.

*P.aeruginosa* was stained with 10 mM CFSE (Sigma) for 3 h and then washed twice to remove excess stain. These cells and WT *S*. Typhimurium were mixed in 1:1 ratio amounting to a total of 10^8 cells.

### Confocal Microscopy: Sample Preparation and Imaging

Strings post staining and washing were placed on a glass coverslip and fixed using 4% PFA for 30 min at room temperature. Excess PFA was removed by gentle washing with PBS. 1% DABCO was added as the anti-fade. Imaging was done using Leica SP8 confocal microscope and images were analyzed using Fiji (Schindelin et al., 2012).

### Atomic Force Microscopy: Sample Preparation and Imaging

Strings isolated at 10 h were washed three times with sterile MilliQ water to remove any residual media components. The strings were then placed onto a poly-L-lysine coated glass coverslip. The samples were then air-dried at room temperature in a laminar flow hood for 1 h. The non-adherent bacteria were removed by washing the coverslips once with MilliQ water. These were again air-dried for 1 h. AFM imaging was performed as previously reported (Bhaskarla et al., 2016; Verma et al., 2018).

### Plasmid Construction

*bcsA* was cloned under *trc* promoter in pTrc99a plasmid. The gene encoding *bcsA* was amplified from WT 14028s genomic DNA using Phusion polymerase using appropriate primers (Table 2). The amplified DNA was digested using Xba1 and Sac1 and ligated into the pTrc99a vector digested with the same enzymes to yield the plasmid p*bcsA* and verified using sequencing.

### c-di-GMP Detection and Quantitation Using HPLC

The protocol for c-di-GMP extraction and quantitation was the one previously reported (Petrova and Sauer, 2017). 1 ml of 2 OD cells and strings isolated from 20 ml of culture were used for this assay. Post extraction the c-di-GMP containing pellet was resuspended in 200 μl of nanopure water just before use (stored at −80°C). 20 μl was injected and analyzed using HPLC (Thermo Finnigan Surveyor HPLC) and compared to standard plots generated with sodium salt of c-di-GMP (HPLC grade, Sigma) at concentrations 1, 2, 5, 10 and 20 pmol. The values of c-di-GMP detected were then normalized for protein content.

### Biofilm Production and Quantitation

Biofilms were generated in 12 well plastic plates (Tarsons, India) in LB minus NaCl, M9, M9 + 200 μM 2,2- Bipyridyl. 50 μl of 2 OD cells were inoculated in 1.5 ml media and kept at 30°C for 5 days in static condition. Biofilms were quantitated using 1% (w/v) crystal violet staining and 33% (v/v) acetic acid extraction and the absorbance was measured at 570 nm (Ray et al., 2020; Thakur et al., 2020).

### RNA Purification and cDNA Synthesis

Total RNA was extracted from WT cells and strings after 10 h using TRIzol reagent (Sigma, St. Louis, MO, United States), according to manufacturer’s instructions. The pellet was dried and dissolved in 25 μL Nuclease free water (NEB, MA, United States). RNA concentration and purity were quantified using NanoDrop Spectrophotometer (Thermo Scientific, Waltham, MA, United States). This was followed by DNase treatment of 1 μg of RNA using a Turbo DNA free kit (Invitrogen, United States) as per manufacturer’s instructions. Subsequently, 500ng of RNA was reverse transcribed to cDNA using RevertAid (Thermo Scientific).

### Quantitative Real-Time PCR

cDNA was diluted 1:20 and analyzed using BioRad CFX-Connect Real Time PCR Detection system (BioRad, CA, United States) with SYBR Green I detection. Each sample was measured in triplicate in a 96-well plate (BioRad) in a reaction mixture (10 μL final volume) containing 2X SYBR iQ SYBR Green supermix and 10 μM primer mix (Supplementary Table 2). The threshold cycles (Ct) were calculated using the iQ5 Optical system software, and fold changes normalized to the reference control *rrlC* were determined using the 2^–ΔΔCt^ method (Ray et al., 2020).

## Results

### Strings Are Formed Under Bipyridyl Stress in Minimal Media

**FIGURE 1:**
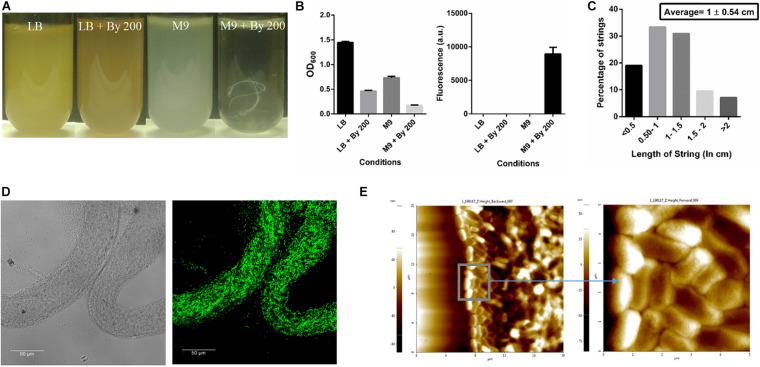
*Salmonella*Typhimurium under Bipyridyl stress in minimal medium produces string-like structures. **(A)** Representative images of test tubes of Salmonella Typhimurium after 10 h at 37°C under 180 rpm in different growth conditions: Nutrient rich media (LB), Nutrient rich media with By 200 (200 μM 2,2 Bipyridyl, Fe2+ chelator) (LB + By 200), Minimal media (M9) and Minimal media with By 200 (M9 + By 200/M9 + Bipd) containing 0.4% (w/v) glucose as the carbon source, with white string like structures clearly visible **(B)** Quantification of strings formed using SYTO9 (nucleic acid stain, staining both live and dead Salmonella cells) after through washing to remove planktonic cells and planktonic growth under various conditions (*n* = 3) **(C)** Histogram depicting distribution of string length with average ± SD (*n* > 40) under M9 + By 200 conditions **(D)** Confocal Images of a strings formed by cells harboring GFP expressing plasmid at 63X (scale bar 50 μm) **(E)** Atomic Force Microscopy (AFM) imaging of strings to study arrangement of cells with 4X magnification of inset (20 μm and 5 μm squares). Data is represented as mean ± SEM.

Initially, strings formed by *Salmonella* Typhimurium 14028s were observed when 200 μM of 2,2-Bipyridyl (Bipd) was added to M9 medium with 0.4% (w/v) glucose as carbon source. We observed around 1-3 strings per 5 ml of media. A protocol was then designed, after optimizing dose and time points, to quantify strings using SYTO9 a nucleic acid stain which binds DNA (Figures 1A,B, Supplementary Figures 1, 2). SYTO9 stains both live and dead *Salmonella* Typhimurium and has been used for biofilm quantitation (Berney et al., 2007; Peeters et al., 2008). Extensive cell-cell contact due to the rotating motion of the vessel containing the culture was required for string formation and a larger proportion of cells entered the string with increasing RPM as shown by the decreasing normalized OD with increasing RPM (Supplementary Figures 2C,D). After calibrating SYTO9 fluorescence with known number of single cells, we found that a single string on average contains nearly a billion cells (Supplementary Figure 3). These structures were, however, not formed upon addition of Bipd to LB (Figures 1A,B). Strings were also found to have an average length of ∼ 1 cm (Figure 1C). Confocal imaging of strings formed by GFP positive *S.* Typhimurium demonstrated that strings were composed of cells, but they were not packed in any discernible pattern (Figure 1D) and confirmed by AFM imaging (Figure 1E). To rule out strain specific effects, string formation was studied in the *S.* Typhimurium SL1344 strain in M9 containing 200 μM Bipd (M9 plus Bipd). *S.* Typhimurium SL1344 also formed strings in similar amounts to the 14028s strain, demonstrating that string formation was not strain specific (Supplementary Figure 4).

### Multi-species Strings Can Also Be Formed

We studied if other bacterial species could also form strings. *E. coli* MG1655 did not form macroscopic strings under Bipd stress although the planktonic growth was similar to *S*. Typhimurium (Supplementary Figure 4). We then studied whether multi-species strings could be formed like multi-species biofilms (Galperin, 2018). When both *E. coli* and *S.* Typhimurium were inoculated in 1:1, multi-species could be formed, suggesting *E. coli* could become a part of *Salmonella* strings (Supplementary Figure 5A). Although *Pseudomonas aeruginosa* did not grow well in M9 alone itself (data not shown), it could also become part of multi-species strings when *P. aeruginosa* and *S.* Typhimurium were inoculated in a 1:1 ratio (Supplementary Figure 5B).

### String Formation Helps Bacteria Evade Bacteriostatic Effects of Bipd

**FIGURE 2:**
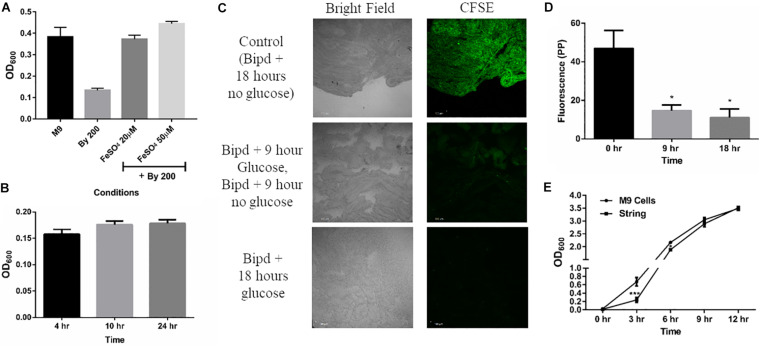
String-like structure help in the evasion of Bipyridyl induced bacteriostasis. **(A)** Planktonic growth in M9 + 200 μM 2,2-Bipyridyl upon supplementation with Fe^2+^ by adding fresh FeSO_4_ at indicated concentrations (*n* = 3). **(B)** Planktonic growth of cells in M9 + Bipd at the varying time points (*n* = 3). **(C)** Imaging of Strings pre-stained using CFSE (Carboxyfluorescein succinimidyl ester, stains cell proteins) after 18 h in M9 + Bipd with or without C source (glucose at 0.4% w/v) for indicated time points to visualize cell division via reduction of CFSE fluorescence. Control of strings in media with no glucose for 18 h to account for reduction in CFSE fluorescence due to bleaching. Images at 20X (scale bar 100 μm) (*n* = 3). **(D)** Quantitation of CFSE fluorescence to detect protein synthesis and cell division * = *p* < 0.05, One-way ANOVA from images in 2C **(E)** Planktonic growth emanating from strings harvested from M9 + Bipd and control planktonic M9 cells (Starting at 10^7 CFU/ml) upon removal of Bipd stress and entering nutrient rich media (LB) at varying time points. * = *p* < 0.05, *** = *p* < 0.005 Two-way ANOVA. Data is represented as mean ± SEM.

Next, we studied whether string formation would help in evasion of the inducing stress. Bipd has been shown to exert a bacteriostatic effect due to chelation of Fe^2+^, an essential micronutrient. (Jurado, 1997; Abdul-Tehrani et al., 1999). First, we verified that the Bipd effect was dependent on Fe^2+^ chelation: the addition of fresh FeSO_4_ to minimal media containing Bipd restored planktonic growth to levels observed in M9 alone (Figure 2A). We also observed a reduction in string formation with the addition of FeSO_4_, we could not quantify it due to conversion of soluble Fe^2+^ to insoluble Fe^3+^ salt precipitates which could not be washed off from strings. We then verified that Bipd exerted a bacteriostatic effect on planktonic cells by observing no significant increase in growth from 4h to 24h (Figure 2B). If strings could evade the bacteriostatic effect, then cells inside the string would divide. Carboxyfluorescein succinimidyl ester (CFSE) is a protein staining dye, exhibiting fluorescence properties similar to GFP, that is routinely used to study cell division in various immune cells and has been used for bacteria as well (Lyons, 2000; Vander Top et al., 2006). The method is based on initially prestaining cells with CFSE which is non-toxic. As cells grow, the protein content increases and gets divided into the daughter cells. Therefore, the CFSE dye bound to the proteins would also get divided between the daughter cells (Lyons, 2000). Thus, dilution of CFSE dye acts as a proxy for measuring cell growth and division. To study if strings could evade Bipd’s bacteriostatic effect, we stained strings (6h old formed in M9 + Bipd) with CFSE and then transferred them to fresh M9 + Bipd in the absence or presence of 0.4% (w/v) glucose. M9 + Bipd not containing glucose acted as a control to ensure reduction in CFSE fluorescence was not due to bleaching or decay of the dye. The CFSE fluorescence intensity in M9 + Bipd containing glucose was significantly lower in both 9 hr and 18 hr time points compared to the control, suggesting that cells in the strings divided and were able to evade the bacteriostatic effects of Bipd (Figures 2C,D). To verify whether string formation was a stress response, we inoculated washed strings into fresh nutrient rich LB media and investigated whether cells in strings could revert to a planktonic state upon reversion to favorable conditions. We reasoned that if cells came together to form strings to evade stress, they would be able to revert back upon removal of stress inducing conditions. We observed that cells in the strings did revert to a planktonic state, since the OD data reached comparable levels of the inoculum of ∼1 billion planktonic cells (grown in M9) by 6 h (Figure 2E).

### String Formation Confers Protection Against Various Stresses That Induce It as Well as Antibiotics

**FIGURE 3:**
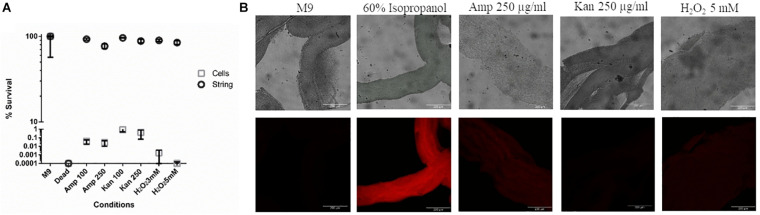
Strings confer protection against high doses of antibiotics and oxidative stress **(A)** The percentage survival of washed strings formed in M9 + Bipd and planktonic cells in M9 after 3 h of treatment under 8-20 fold MBC (Minimum Bactericidal Concentration) of ampicillin, kanamycin or oxidative stress individually at multiple doses in fresh M9. Viability of strings determined by PI staining and planktonic cells by plating (instead of PI due to single cell lysis). (60% Isopropanol treatment kills all cells in the string, Error for dead (strings) is 7.53%, kanamycin (Kan) and ampicillin (Amp) in μg/ml) (*n* = 3). **(B)** Confocal images of PI stained strings post varying stresses for 3 h taken at 20X (scale bar 200 μm) (*n* = 3). The data is represented as mean ± SEM.

Next, we studied whether strings, like biofilms, were equipped to resist higher doses of antibiotics like ampicillin and kanamycin and other stresses such as oxidative stress, compared to planktonic cells (Mah and O’Toole, 2001; Høiby et al., 2010; Kaplan, 2011) To study the death induced by various stresses in strings, we used Propidium Iodide (PI) staining and then measured relative fluorescence intensity of each treatment group between untreated strings kept in fresh M9 and strings killed with 60% isopropanol to quantify the percentage of dead cells in strings. Planktonic cells subjected to similar stresses were plated on SS agar plates and CFU/ml was calculated and compared to the same controls. PI staining was not used for the planktonic cells since both ampicillin and kanamycin would lyse the planktonic cells (Heller and Spence, 2019). Strings were more resistant to higher doses of both bactericidal antibiotics tested, e.g., Ampicillin and Kanamycin (> 100 fold) compared to planktonic cells (Figures 3A,B). In oxidative stress, strings were more resilient and showed > 1000-fold survival compared to planktonic cells (Figure 3A).

### String Formation Is Not Related to Classical CsgD-Dependent Biofilms

**FIGURE 4:**
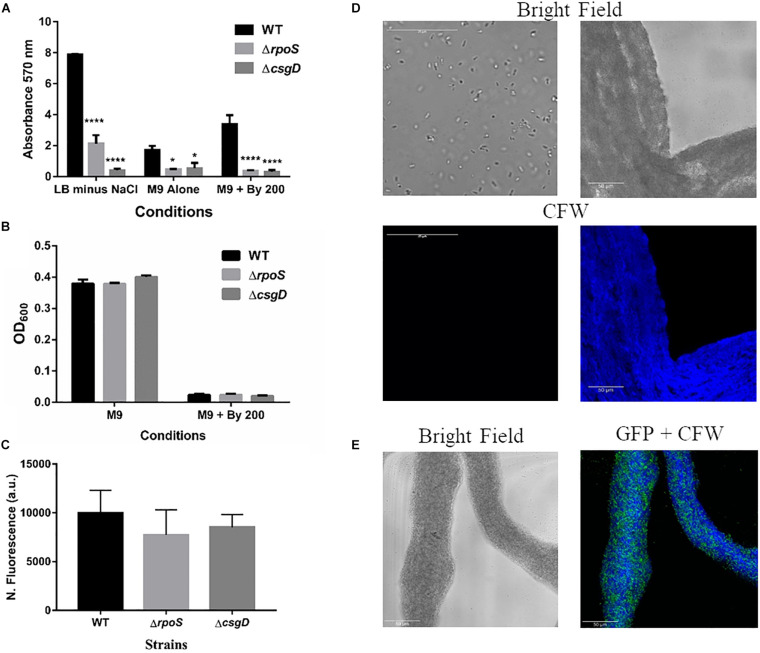
String formation is independent of key molecules required for biofilms but show increased cellulose. **(A)** Biofilm formation by WT, ΔrpoS and ΔcsgD under various conditions at day 5 time point in 30°C without shaking. * = *p* < 0.05, **** = *p* < 0.001, Two-way ANOVA, **(B)** Planktonic growth in WT, ΔrpoS and ΔcsgD strains in M9 or M9 + Bipd (*n* = 4). **(C)** String formation in M9 + Bipd by WT, ΔrpoS and ΔcsgD. **(D)** Images of cells from M9 and strings formed in M9 + Bipd (63X) after Calcofluor White (CFW) staining to detect cellulose. Bright field on upper half and after UV excitation in the lower half. **(E)** Represents CFW stained string formed by GFP positive cells to show cellulose encasement of cells. The scale bar is 25 μm for planktonic cells and 50 μm for strings. The data represented as mean ± SEM.

Given that *S.* Typhimurium forms robust biofilms at surfaces/interfaces, it was important to address if strings are related to classical CsgD-dependent biofilms. The approach was to determine whether conditions that induce these biofilms could also induce strings and vice-versa. While low osmolarity (LB minus NaCl) condition produced robust biofilms, it did not give rise to strings. Also, ethanol which also led to strings in M9, did not induce biofilm formation while giving robust string formation (Supplementary Figures 6B,C). Further verification was performed using mutants known to be defective in classical CsgD-dependent biofilm formation, e.g., strains lacking CsgD or RpoS. As expected, the Δ*csgD* and Δ*rpoS* strains were defective in biofilm formation in the LB minus NaCl, M9 and M9 plus Bipd conditions compared to WT (Grantcharova et al., 2010; Mika and Hengge, 2014; Figure 4A). However, string formation was similar in all three strains (Figures 4B,C). These two observations together demonstrate that mechanisms involved in classical CsgD-dependent biofilm formation and string formation in *Salmonella* are distinct.

### Cellulose Production Is Critical for String Formation

**FIGURE 5:**
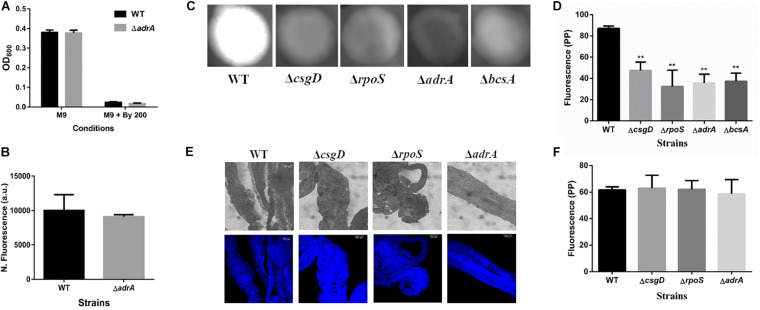
Cellulose production in strings is independent of CsgD. **(A)** Planktonic growth in WT and ΔadrA strains in M9 or M9 + Bipd (*n* = 4). **(B)** String formation in M9 + Bipd by WT and ΔadrA (*n* = 4). **(C)** Cellulose production in WT, ΔbcsA, ΔcsgD and ΔrpoS mutants in LB minus NaCl agar determined by Calcoflour staining after 48 h at 30°C. **(D)** Quantitation of fluorescence as a measure of cellulose production in colonies, ** = *p* < 0.01, *** = *p* < 0.005, One-way ANOVA. **(E)** Calcofluor White staining of strings formed by various mutants (Images at 20X), scale bar 100 μm. **(F)** Quantitation of fluorescence as a measure of cellulose production in strings. The data represented as mean ± SEM.

**FIGURE 6:**
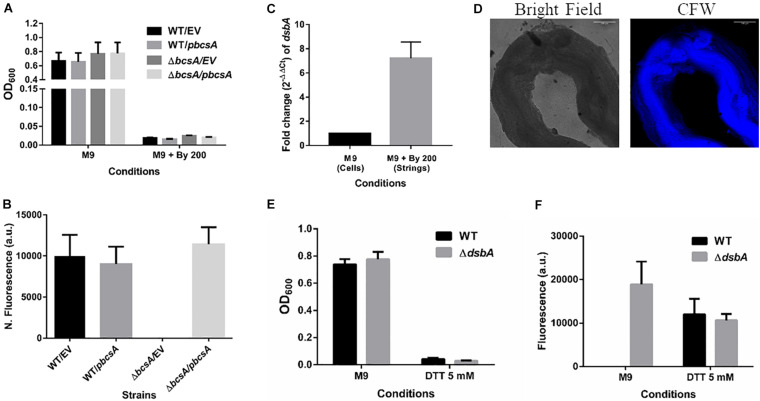
Cellulose is critical for string formation and reductive stress is sufficient to activate string formation. **(A)** Planktonic growth of WT and ΔbcsA strains complemented with Empty Vector (pTrc99a) or pbcsA. (*n* = 4). **(B)** Quantitation of string formation in the four strains in M9 + Bipd. **(C)** The fold difference in transcript levels for dsbA in cells from M9 and strings formed under M9 + Bipd at 10 h time point (*n* = 4). **(D)** Images of Calcofluor White stained strings formed under dithiothreitol (DTT) 5 mM stress (20X) in bright field and after UV excitation to detect cellulose production. The scale bar is 100 μm. **(E)** Planktonic growth in M9 and 5 mM DTT of WT and ΔdsbA mutant. **(F)** Quantitation of string formation in WT and ΔdsbA mutant in M9 and 5 mM DTT stress. (*n* = 4). The data represented as mean ± SEM.

It was important to identify the matrix involved in packing of cells within strings. An extracellular matrix comprising of cellulose could encase cells and lead to the formation of strings. Staining with Calcoflour white, a cellulose binding dye, demonstrated higher fluorescence for strings compared to planktonic cells grown in M9 (Figure 4D). Imaging strings formed by GFP positive *S.* Typhimurium after Calcoflour staining, clearly showed that cells were encased in a cellulose matrix (Figure 4E). To further confirm the roles of cellulose, a deletion strain of a cellulose synthase complex component *bcsA* was generated. The Δ*bcsA* strain did not form strings suggesting that cellulose was critical for string formation (Figures 5A,B), although the Δ*bcsA* strain was not defective in biofilm formation (Supplementary Figure 6A). Further, complementing the Δ*bcsA* strain with WT *bcsA*, but not the empty vector control, restored its ability to form strings. Notably, the planktonic growth remained similar in all four strains (Figure 6A). These results clearly demonstrate a key role of cellulose in string formation.

Next, we studied if strings formed by the mutants Δ*csgD*,Δ*rpoS* and SL1344 strain were also dependent on cellulose. As previously reported (García et al., 2004; Mika and Hengge, 2014; Römling and Galperin, 2015), these strains were defective in cellulose production in LB minus NaCl agar model as determined by Calcoflour staining and was comparable to the Δ*bcsA* strain. (Figures 5C,D, Supplementary Figures 7A,B). However, we observed that strings formed by all these strains produced equal amounts of cellulose based on Calcoflour staining, further demonstrating that cellulose is critical for string formation. Importantly, this observation also suggested a mode of regulation of cellulose production independent of the classical CsgD-dependent biofilm pathway (Figures 5E,F, Supplementary Figures 7C,D). Cellulose production independent of CsgD is known in both *E. coli* and *Salmonella* (Da Re and Ghigo, 2006; Hufnagel et al., 2014; Simm et al., 2014). The SL1344 strain is defective in cellulose production due to a deficiency in MlrA which controls the expression of CsgD (García et al., 2004; Römling and Galperin, 2015) and further confirms our results that cellulose production in strings is independent of CsgD.

### Strings Are Also Induced by Some Stresses Including Antibiotics in a Cellulose Dependent Manner

Next, we investigated whether other stress could also induce string formation. Interestingly, we found that other stresses such as chloramphenicol at sub- optimal doses (5 μg/ml) and ethanol [2–5% (v/v)] also induced string formation (Supplementary Figure 8A). However, others like oxidative stress (Supplementary Figure 8A), pH (4–10), bile (1–5% w/v), and detergents like SDS (0.2– 1% w/v), Tween 20 (0.5–2% v/v) induced stress did not form strings in both M9 and LB (data not shown). Taken together, these results suggest that string formation is induced by some, but not all, stress conditions. We further investigated whether string formation in other stresses were also dependent on cellulose production. Interestingly, strings formed under the stresses of chloramphenicol and ethanol, also stained positive for cellulose while cells exposed to oxidative stress that did not form strings stained negative (Supplementary Figure 8B). Next, we studied whether the Δ*bcsA* strain could give rise to strings in these conditions. We observed that Δ*bcsA* strain was defective in string formation in both these stresses and upon complementation with WT *bcsA* string formation was restored, proving that cellulose is critical for string formation across varying conditions (Supplementary Figure 8C).

### Reductive Stresses Also Induce String Formation

CsgD-independent cellulose production in both *E. coli* and *Salmonella* has been shown to be activated by reductive stress conditions (Hufnagel et al., 2014; Simm et al., 2014). To test whether Bipd in minimal media induced reductive stress via chelation of Fe^2+^ that can contribute to ROS via Fenton reaction, we studied *dsbA* transcript levels in the cells. The disulfide bonding (DSB) system is involved in oxidizing reduced proteins, and an increase in *dsbA* and *dsbB* is expected when cells face reductive environments (Gutteridge et al., 1990; Bardwell et al., 1993). Compared to M9, M9 + Bipd cells had 6-fold higher levels of *dsbA* suggesting that Bipd does induce reductive stress (Figure 6C). To directly determine the role of reductive stress, we tested whether DTT alone in minimal media would also lead to string formation in a cellulose dependent manner. We observed that DTT did induce string formation in a cellulose-dependent manner. (Figures 6D–F). Next, we confirmed the role of reductive stress by observing string formation in M9 alone for the Δ*dsbA* strain, which would intrinsically have a higher reduced state (Bardwell et al., 1993; Hufnagel et al., 2014) (Figures 5E,F). Together, these studies unequivocally demonstrate that reductive stresses in minimal media are important for string formation.

### Strings Formation Is Dependent on the Bacterial Secondary Messenger c-di-GMP

**FIGURE 7:**
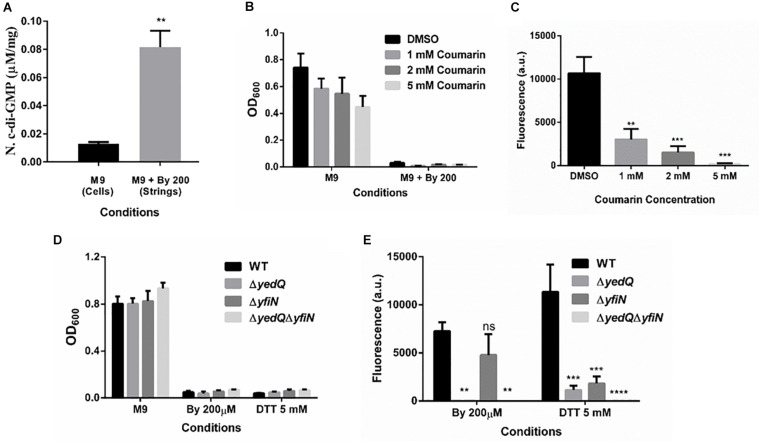
Increase in levels of bacterial secondary messenger c-di-GMP via diguanylate cyclases YedQ primarily and YfiN is critical for string formation. **(A)** c-di-GMP levels in cells in M9 vs strings formed in M9 + Bipd at 10 hr measured by using HPLC (*n* = 3) and normalized using protein levels determined by Bradford assay. ** = *p* < 0.01, Students *T*-test **(B)** Effect of Coumarin a molecule known to reduce c-di-GMP levels in cells, on planktonic growth in M9 and M9 + Bipd. **(C)** Effect of Coumarin on String formation in M9 + Bipd. **(D)** Planktonic growth of WT, ΔyedQ, ΔyfiN and ΔyedQΔyfiN mutants under various conditions. **(E)** String formation of WT, ΔyedQ, ΔyfiN and ΔyedQΔyfiN mutants under various conditions ** = *p* < 0.01, *** = *p* < 0.005 **** = *p* < 0.001, Two-way ANOVA. The data is represented as mean ± SEM.

Cellulose production through the cellulose synthase complex is controlled by c-di-GMP (Römling, 2002; Römling and Galperin, 2015). Therefore, we performed an HPLC analysis to quantify c-di-GMP levels in strings compared to M9 planktonic cells. We observed a 6-fold increase in c-di-GMP levels in strings under Bipd stress compared to planktonic cells in M9 (Figure 7A). To confirm the roles of c-di-GMP, we utilized Coumarin, a compound that has multiple effects but also importantly reduces intracellular c-di-GMP amounts (Zhang et al., 2018; Thakur et al., 2020). Treatment with Coumarin decreased string formation in M9 plus Bipd extensively but not planktonic growth, suggesting a potential role for c-di-GMP during string formation (Figures 7B,C).

### Diguanylate Cyclases YedQ and YfiN Mediate String Formation

c-di-GMP production in *Salmonella* is mediated by the major DGCs, AdrA and YedQ; therefore, we tested whether mutants for either would abrogate string formation (Römling and Galperin, 2015). We found the *adrA* deletion had no effect on string formation (Figures 5A,B). The Δ*adrA* strain, however, did reduce cellulose levels in the LB minus NaCl growth conditions, while strings of the Δ*adrA* strain contained cellulose amounts similar to WT strings (Figure 5). String formation was, however, completely abrogated in the Δ*yedQ* mutant in M9 + Bipd conditions while in M9 + DTT the mutant was significantly less efficient compared to WT. (Figures 7D,E). Given that YfiN also contributes to c-di-GMP production in reductive environments in *E coli* and had been shown to substitute YedQ in ATM media, we constructed a Δ*yfiN* single and Δ*yfin*Δ*yedQ* double mutant to test their roles (Solano et al., 2009; Hufnagel et al., 2014). The single *yfiN* deletion lowered string formation in M9 + DTT to the same extent as *yedQ* but the effect in M9 + Bipd was only modest. (Figures 7D,E). However, the Δ*yfin*Δ*yedQ* mutant did not form strings in both M9 + Bipd and M9 + DTT conditions. (Figures 7D,E). This data demonstrates that string formation is mediated primarily by YedQ and partly by YfiN.

## Discussion

Filamentous forms of bacteria, small aggregates like biofilms, multi-cellular magneto-tactic prokaryotes, *Mycobacterial* cording, *Rhodobacter* clumping, *Zymomonas* aggregates are the main multi-cellular structures previously reported in bacteria (Puskas et al., 1997; Shapiro, 1998; Rudolph et al., 2001; Bernut et al., 2014; Lyons and Kolter, 2015; Jones-Burrage et al., 2019). In this study, we report a novel macroscopic multi-cellular structure formed by *Salmonella* Typhimurium referred to as “strings” which are ∼ 1 cm in size and contain around a billion tightly packed cells. (Figure 1, Supplementary Figure 3). In addition, we show that strings and biofilms are unrelated, and that string formation requires high amounts of cellulose production in liquid culture. This high cellulose production is mediated by increased c-di-GMP production via DGCs, YedQ primarily and YfiN partly. Extensive cell-cell contact brought by shaking conditions were required to form strings. This suggested that upon cellulose production by individual cells, they must come in contact and the shear force/drag drives cells to form structures as large as a string (Supplementary Figure 2D). In the absence of shaking conditions, cells may clump after settling or form smaller aggregates similar to cords formed by *Mycobacterium.* Lower planktonic growth under some stress conditions correlated with string formation suggesting that cells become part of the growing strings instead of remaining as single cells (Supplementary Figure 2C). In addition, we show that multi-species strings can be formed by *S.* Typhimurium with *E. coli* or *P. aeruginosa* (Supplementary Figure 5). Further studies are required to identify the ability of other bacteria, especially pathogenic bacteria, to form strings and characterize them under different conditions. How the host environment may induce formation of strings in pathogenic bacteria and their ability in resisting antibiotics or immune responses *in vivo* is an area that need to be further investigated.

To investigate the relationship between strings and classical CsgD-dependent biofilms, we studied whether conditions inducing strings induce biofilms and vice-versa. We observed that conditions inducing biofilms do not necessarily result in strings in shaking conditions and vice-versa, which suggested that biofilm and string formation are distinct (Supplementary Figure 6). Interestingly, some of the key genes like *csgD* and *rpoS* which are involved in biofilm formation (Mika and Hengge, 2014; Simm et al., 2014) do not play critical roles in string formation (Figure 4). The observation that the Δ*csgD* strain forms strings suggests that major extracellular polymers like curli fimbriae, O antigen capsule etc, whose transcription is directly regulated by CsgD may not be important for string formation (Mika and Hengge, 2014). The fact that Δ*bcsA* also formed biofilms by day 5 (Supplementary Figure 6A) is supported by the fact that curli is the major component of biofilms and that lack of *bcsA* only affects initial biofilm formation (Grantcharova et al., 2010). All these observations together suggest that string formation is independent of the classical CsgD-dependent biofilms.

Previous reports of CsgD-independent cellulose production pointed to reductive stresses playing a role (Hufnagel et al., 2014). To investigate whether reductive stresses led to an increase in string formation, we added DTT into minimal media and scored for string formation. As seen in Figures 6D–F, DTT-induced reductive stress led to string formation in a cellulose-dependent manner. Addition of Bipd leading to a 6-fold increase in *dsbA* levels suggested that Bipd also induces a reducing stress possibly via Fe^2+^ chelation (Figure 6C) (Gutteridge et al., 1990; Bardwell et al., 1993). We also observed that just deleting *dsbA* led to string formation in minimal media alone under no other stress (Figures 6E,F). Overall, these results demonstrate that reductive stress along with nutritional stress is sufficient to induce string formation (Figure 6).

**FIGURE 8:**
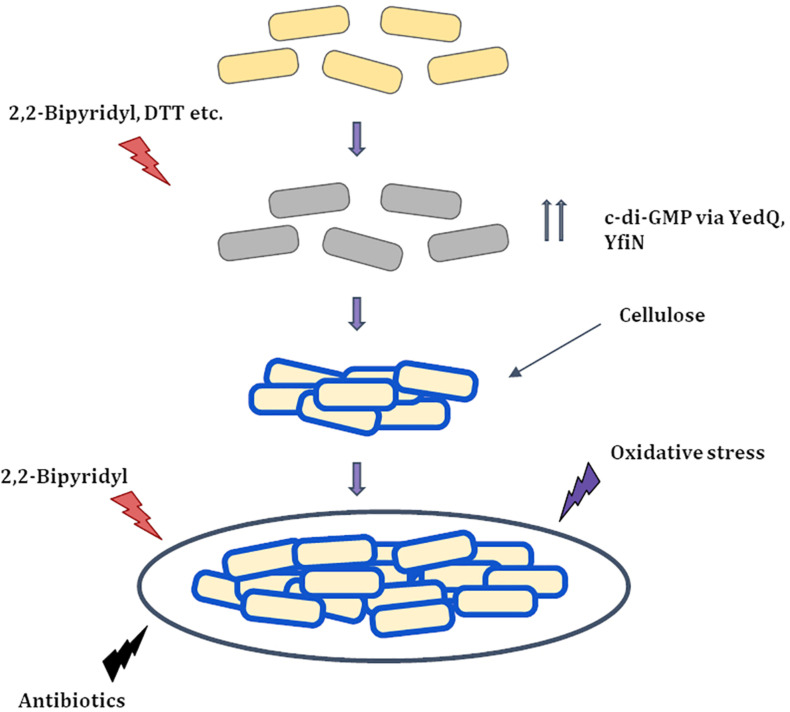
Proposed Model for string formation (i) Cells upon exposure to 2,2-Bipyridyl or Dithiothreitol (DTT) increase the production of c-di-GMP via DGC’s YedQ and YfiN to produce cellulose. (ii) The cells surface gets coated by cellulose and the shaking condition causes them to cluster and form strings. (iii) String formation and cellulose coating leads to greater resistance to stress conditions that induced them and others like antibiotics and oxidative stress. 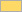 Healthy cells 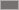 Stressed cells 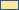 Partially recovered cells with cellulose coating (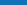).

The basic mechanism for string formation appears to be increased production of the bacterial secondary messenger c-di-GMP which regulates cellulose production (Römling, 2002; Römling and Galperin, 2015; Figure 8). Coumarin a molecule known to reduce c-di-GMP levels at the concentrations used, lowered planktonic growth marginally while greatly reducing string formation (Figures 7B,C). This suggests that Coumarin reduced string formation by lowering c-di-GMP levels in strings. However, coumarin could also have other targets like quorum sensing, type III secretion system as well, raising the possibility that other factors may also be involved in this reduction (Zhang et al., 2018; Thakur et al., 2020). Strings stained positive for cellulose as judged by calcofluor staining but not planktonic single cells in M9. The role of cellulose was confirmed by constructing a Δ*bcsA* strain which did not form strings in M9 + Bipd and upon complementing with WT *bcsA* restored string formation (Figure 6). String formation under all other conditions tested relied on cellulose production whereas cells exposed to oxidative stress which did not stain positive for cellulose and did not induce strings (Supplementary Figure 8). The critical role of cellulose could explain why *E. coli* MG1655 did not form strings, since it is defective in cellulose production (Zogaj et al., 2001; Da Re and Ghigo, 2006). Thus, string formation seems to occur due to increased cellulose production during reductive stresses.

In *Salmonella* c-di-GMP production is mediated mainly by DGCs: *adrA* (in a CsgD-dependent manner) or *yedQ* (in a CsgD-independent manner) (Solano et al., 2009; Römling and Galperin, 2015). Given that string formation was independent of CsgD, we found that the Δ*adrA* strain could still form strings. (Figure 5A). Interestingly, mutants of *adrA, csgD, rpoS* and the SL1344 strain all, as expected, produced lesser cellulose compared to WT (Kader et al., 2006; Mika and Hengge, 2014; Römling and Galperin, 2015) and similar to the Δ*bcsA* strain in the LB minus NaCl plate but produce similar amounts of cellulose in strings (Figure 5, Supplementary Figure 7). The SL1344 strain forming strings had indeed suggested that the cellulose production is independent of CsgD, since the strain is deficient in MlrA, leading to reduced CsgD and AdrA levels (García et al., 2004). Since the *adrA* mutant could still form strings, we generated a Δ*yedQ* deletion strain. We saw that the Δ*yedQ* mutant’s ability to form strings was abrogated in M9 + Bipd and greatly reduced in M9 + DTT, suggesting that YedQ plays a critical role in string formation. These results are consistent with our observation that string formation is independent of CsgD regulated AdrA, as suggested by previous studies delineating YedQ and AdrA dependent cellulose production pathways (Kader et al., 2006). The DGC YfiN has also been shown to play a role in producing c-di-GMP under reductive stresses specifically in *E. coli* (Hufnagel et al., 2014). We saw that the *yfiN* single deletion greatly reduced string formation in M9 + DTT while modestly reduced string formation in M9 + Bipd. However, string formation was completely abrogated in the double mutant in both conditions, suggesting that YedQ is the primary c-di-GMP producer and YfiN partially contributes to string formation. YedQ and YfiN being the two DGC’s important for string formation in minimal media agrees with previous reports showing them as the only two DGCs important in minimal media (Solano et al., 2009).

There are reports by Solano et al. (1998) and García et al. (2004) of aggregates by Salmonella in ATM (another minimal media). Strings are unlikely to be similar to those aggregates: strings are formed only during additional stress in minimal media, are not predominantly attached to a surface and require much lower amounts of glucose (4 g/L compared to 20 g/L). Importantly, no aggregates where formed by the WT SL1344 strain in ATM (García et al., 2004); however, the WT SL1344 strain clearly formed strings in the presence of Bipd in M9 (Supplementary Figure 7). Most likely, the increased cellulose production leading to string formation is a bacterial response to evade stress, e.g., the bacteriostatic effect of Bipd or sub-optimal amounts of antibiotics on planktonic cells. Notably, cells within a string divided under these stress conditions (Figures 2C,D). Strings are also more resistant to various bactericidal stresses compared to planktonic cells (Figure 3). The reasons for increased resistance by cells within strings could be cellulose coating, tight packing of cells and possible lower metabolic rate, similar to biofilms (Mah and O’Toole, 2001; Høiby et al., 2010; Kaplan, 2011).

The link between minimal media and cellulose production also is quite interesting as recently reported aggregation of *Zymomonas mobilis* in minimal media, but not nutrient rich media, is dependent on cellulose production (Jones-Burrage et al., 2019). Our data along with previous reports further demonstrate that the CsgD-independent pathway for cellulose production is more prominent in minimal media (Solano et al., 2009). Additionally, we also provide more insights into the CsgD-independent pathway for cellulose production and how two DGCs, YedQ and YfiN, contribute to formation of strings (Figure 8). Furthermore, we observed that the Δ*dsbA* strain formed strings in minimal media but not in nutritionally rich media LB, demonstrating that reductive conditions in combination with nutritional stress are required for string formation. This is the first report to the best of our knowledge where *Salmonella* produces high amounts of cellulose in a liquid culture instead of an interface such as liquid air or solid liquid.

Strings could have at least two potential applications. First, the high amounts of cellulose and the ease as well as their production rate suggest that strings can be used to mass produce bacterial cellulose. This material has significant biomedical applications for making artificial skin, tissues like blood vessels as well as drug delivery (Portela et al., 2019). Current methods to produce bacterial cellulose mainly utilize *G. hansenii* which takes around 7–8 days and hence is time consuming (Shoda and Sugano, 2005; Costa et al., 2017). Strings could potentially solve this problem as mature strings can be formed within 10 h under shaking conditions. Given that both *E. coli* and *Salmonella* produce modified cellulose having phosphoethanoloamine groups, these modifications may have to be removed before use, or genetic manipulations have to be done to prevent the modification (Galperin and Shalaeva, 2018; Thongsomboon et al., 2018). Secondly, multi-species biofilms are being explored for bioremediation since the microbes effective in clearing the contaminants do not tolerate the harsh environment. Being a component of biofilms helps these cells resist these harsh conditions (Singh et al., 2006; Edwards and Kjellerup, 2013). As multi-species strings can also form and strings, like biofilms, can evade various harsh conditions, strings can also be used for bioremediation (Figure 3).

Overall, strings are a novel macroscopic structure made by *Salmonella* which must be explored further to determine their relevance and possibly realize their potential applications. Further studies are required to investigate whether other bacteria form strings and understand the reasons as to why only certain stresses induce string formation and if there are other pathways for string formation. Additional investigations into string formation *in vivo*, identification of other factors that play a role in string formation, stability, function etc. also need to be studied in future.

## Data Availability Statement

The original contributions presented in the study are included in the article/Supplementary Material, further inquiries can be directed to the corresponding author DN (nandi@iisc.ac.in).

## Author Contributions

AV and DN designed the research and analyzed the data. AV, SR, and TV were involved in performing the experiments and AV did majority of experiments. All authors contributed in writing and editing the draft.

## Conflict of Interest

The authors declare that the research was conducted in the absence of any commercial or financial relationships that could be construed as a potential conflict of interest.
