# Supplementary material for: Multicellular String-Like Structure Formation by Salmonella Typhimurium Depends on Cellulose Production: Roles of Diguanylate Cyclases, YedQ and YfiN
Source: Front Microbiol. 2020 Dec 14;11:613704. doi: 10.3389/fmicb.2020.613704 (PMC7769011; doi:10.3389/fmicb.2020.613704)
Supplement: Supplementary file 1 [file Data_Sheet_1.docx]

**Supplementary Figures**

Figure S1


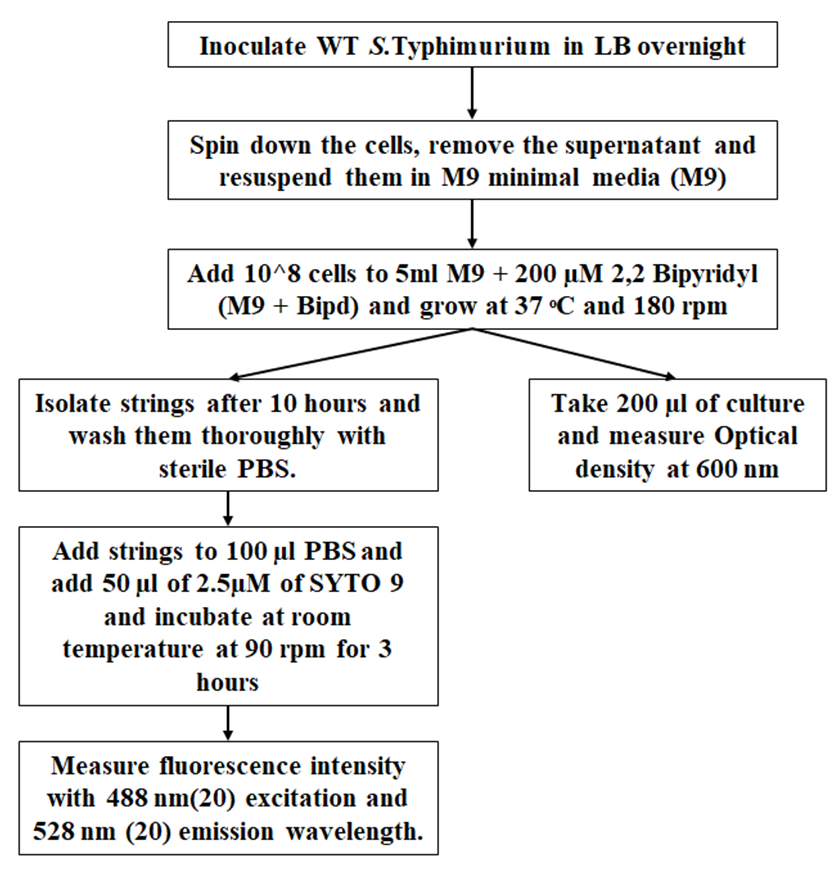


**Figure S1: Flow chart for string formation and quantitation.** After optimizing various steps, the procedure shown in this flow chart was used to study string formation and quantitate them. Strings were handled using cut pipette tips pre-wetted with PBS, to avoid strings sticking to the tips.

Figure S2


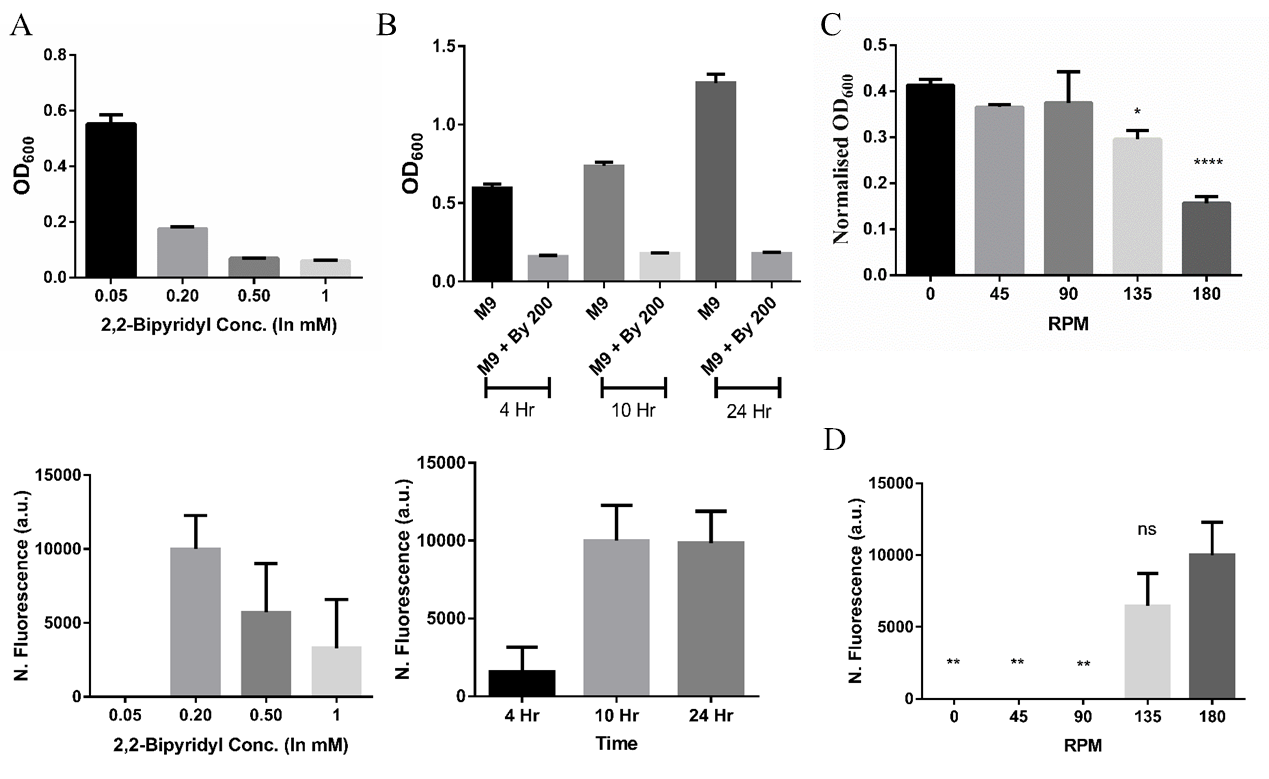


**Figure S2**: **Roles of concentration of 2,2-Bipyridyl, time and RPM on string formation**.(A) Planktonic growth and string formation per 5 ml of culture with in increasing conc. of 2,2-bipyridyl in M9 (n=3) (B) Planktonic growth and string formation per 5 ml of culture with in at varying time points (n=3). (C) Ratio of planktonic growth between M9 + 200 µM 2,2-Bipyridyl and M9 with varying RPM (n=4). *=p<0.05, ****=p<0.001, One-way ANOVA (D) String formation per 5 ml of M9 + 200 µM 2,2-Bipyridyl with varying RPM (n=4). **=p<0.01, One-way ANOVA.. Data is represented as mean ±SEM.

Figure S3


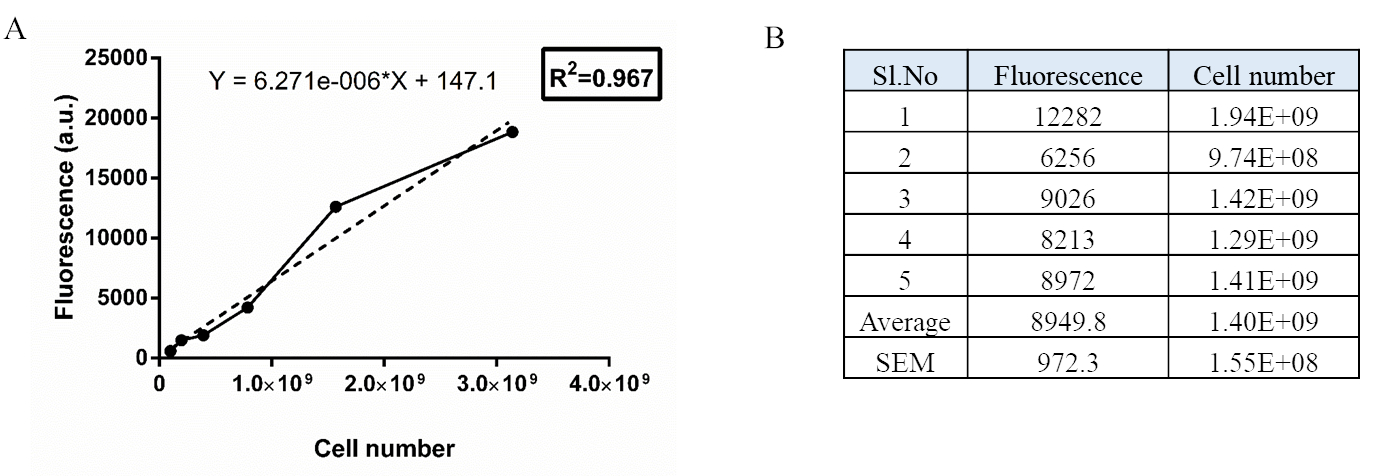


**Figure S3: Quantitation of cell number per string in M9 + 200 µM 2,2-Bipyridyl** (A) Standard curve for fluorescence of known number of planktonic cells after SYTO9 staining (n=3). (B) Table representing cell number per string of approx. 1 cm based on standard curve. Strings were formed in M9 + Bipd and then stained with SYTO9 after through washing.

Figure S4


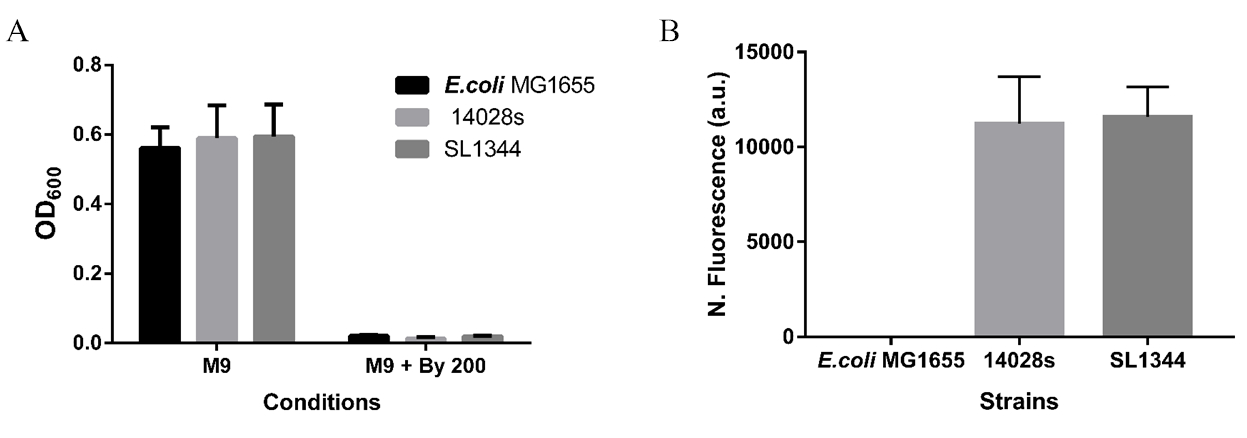


**Figure S4: *S.* Typhimurium SL1344 also forms strings but *E.coli* MG1655 does not.** (A) Planktonic growth (SL1344 supplemented with 20 µg/ml of Histidine) of all 3 strains (n=3) (B) String formation in all three strains (n=3). Data is represented as mean ±SEM

Figure S5


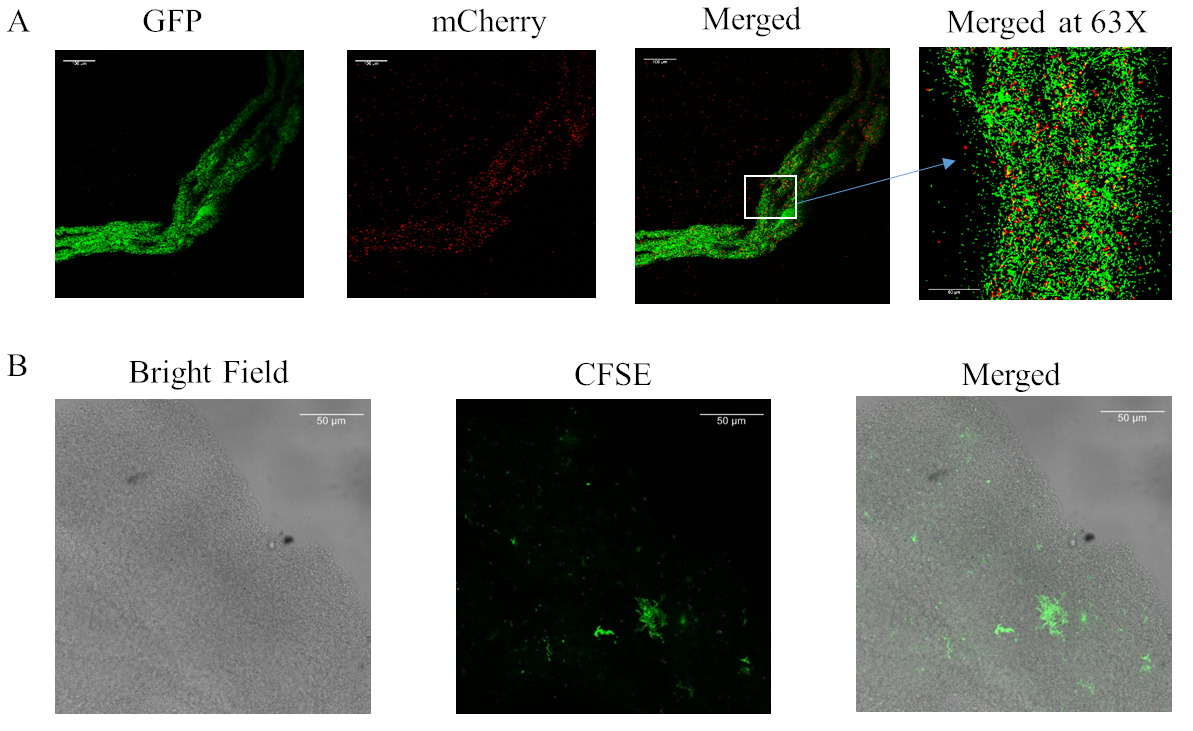


**Figure S5: Multi-species strings formation.** (A) *E.coli* can get incorporated in strings formed by *S.*Typhimurium form multi-species strings (Inoculum in 1:1 ratio of mCherry expressing *E.coli* and GFP expressing *S.*Typhimurium) Images At 20X unless mentioned (scale bar in 20X 100 µm and 63X 50 µm). (B) *P. aeruginosa* can also get incorporated in strings formed by *S.*Typhimurium form multi-species strings (Inoculum in 1:1 ratio of CFSE stained *P. aeruginosa* and WT *S.*Typhimurium 14028s) Images At 20X (scale bar 100 µm).

Figure S6


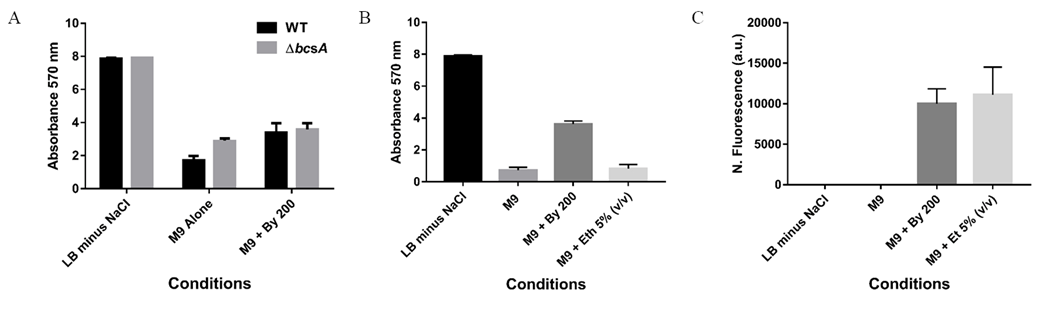


**Figure S6: CsgD-dependent biofilms are not related to strings.** (A) Comparison of biofilm formation by WT and Δ*bcsA* strains across varying conditions**.** (n=3) (B) Biofilm formation under varying conditions (Day 5 on Plastic) (n=3) (C) String formation under varying conditions (At 10 hours) (n=3). Data is represented as mean ±SEM.

Figure S7


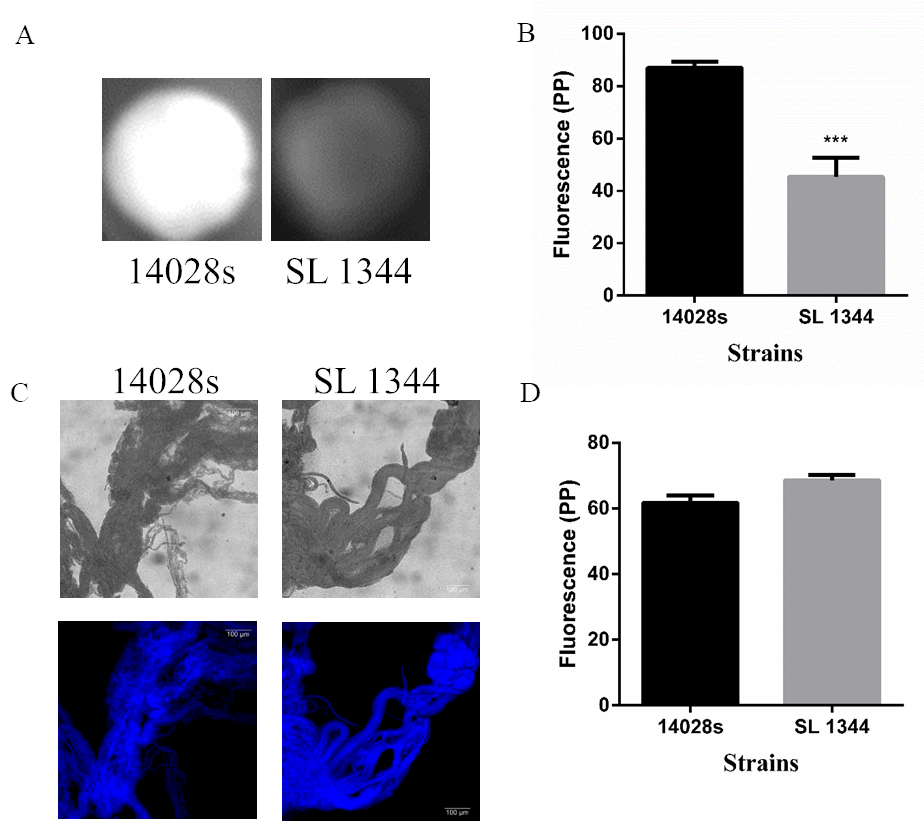


**Figure S7: Cellulose production in 14028s and SL 1344 strains.** (A) Cellulose production between 14028s and in LB minus NaCl agar determined by calcoflour staining after 48 hr. (B) Quantitation of fluorescence as a measure of cellulose production of colonies, ***=p<0.005, One-way ANOVA (C) Calcofluor White staining of strings by both strains (Images at 20X), scale bar 100 µm. (D) Quantitation of fluorescence as a measure of cellulose production in strings. Data is represented as mean ±SEM.

Figure S8


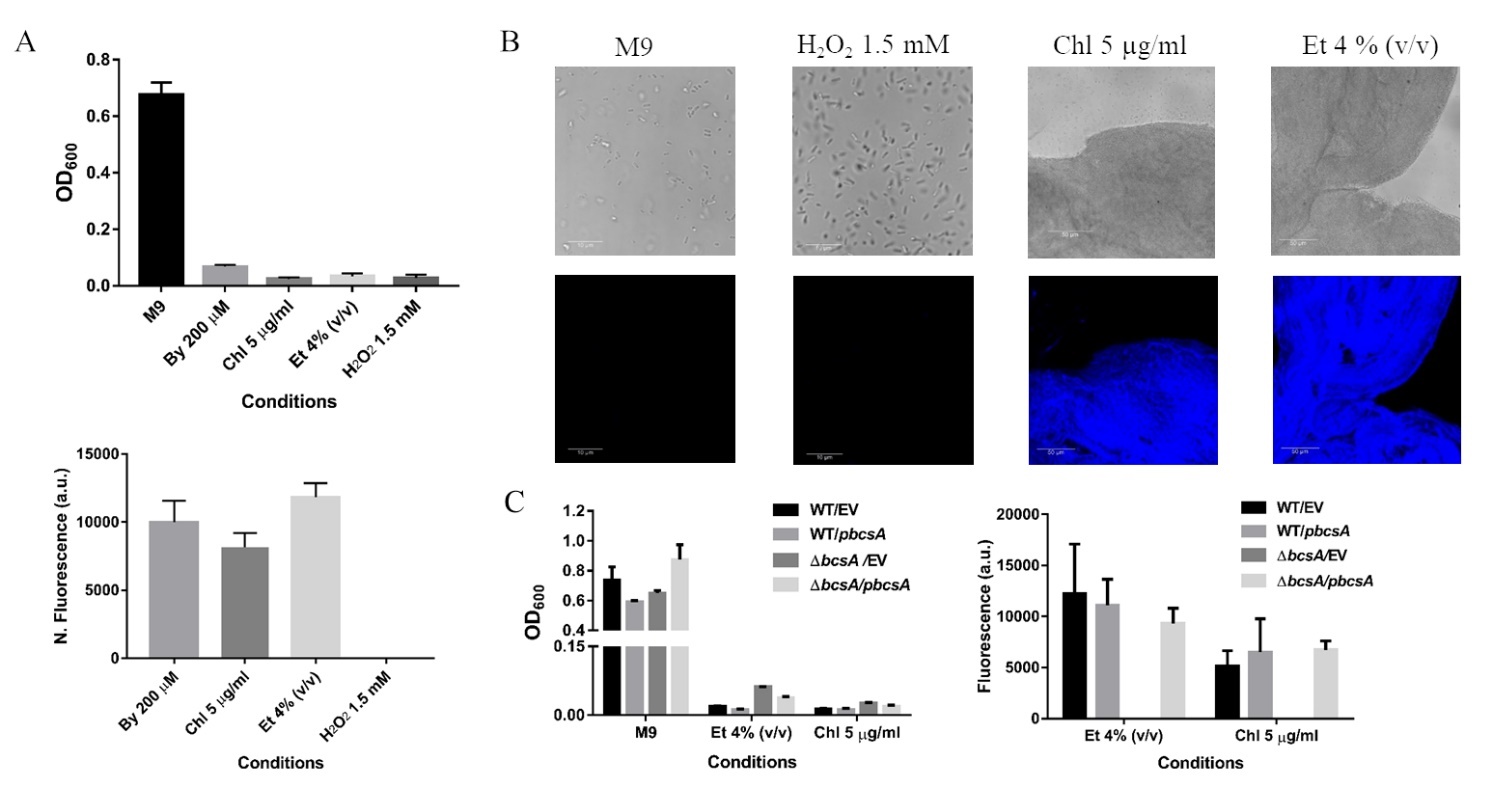


**Figure S8: Cellulose production is critical for string formation in other conditions as well** (A) Planktonic growth and string formation in other stresses as well but not all stresses (Chl in µg/ml, Et in v/v %) (B) Calcofluor White staining of cells or strings from various conditions (63X). The scale bar is 10 µm for planktonic cells and 50 µm for strings. (C) Quantitation of Planktonic growth and string formation of WT and Δ*bcsA* strains complemented with Empty Vector (pTrc99a) or p*bcsA* under varying conditions (n=3). Data is represented as mean ±SEM.
